# Patterns of Convergence and Divergence Between Bipolar Disorder Type I and Type II: Evidence From Integrative Genomic Analyses

**DOI:** 10.3389/fcell.2022.956265

**Published:** 2022-07-15

**Authors:** Yunqi Huang, Yunjia Liu, Yulu Wu, Yiguo Tang, Mengting Zhang, Siyi Liu, Liling Xiao, Shiwan Tao, Min Xie, Minhan Dai, Mingli Li, Hongsheng Gui, Qiang Wang

**Affiliations:** ^1^ Mental Health Center and Psychiatric Laboratory, State Key Laboratory of Biotherapy, West China Hospital of Sichuan University, Chengdu, China; ^2^ West China Brain Research Center, West China Hospital of Sichuan University, Chengdu, China; ^3^ Sichuan Clinical Medical Research Center for Mental Disorders, Chengdu, China; ^4^ Center for Health Policy & Health Services Research, Henry Ford Health System, Detroit, MI, United States; ^5^ Behavioral Health Services, Henry Ford Health System, Detroit, MI, United States

**Keywords:** bipolar disorder, genome-wide association studies, transcriptome-wide association analysis, Mendelian randomization, bipolar type I, bipolar type II

## Abstract

**Aim:** Genome-wide association studies (GWAS) analyses have revealed genetic evidence of bipolar disorder (BD), but little is known about the genetic structure of BD subtypes. We aimed to investigate the genetic overlap and distinction of bipolar type I (BD I) & type II (BD II) by conducting integrative post-GWAS analyses.

**Methods:** We utilized single nucleotide polymorphism (SNP)–level approaches to uncover correlated and distinct genetic loci. Transcriptome-wide association analyses (TWAS) were then approached to pinpoint functional genes expressed in specific brain tissues and blood. Next, we performed cross-phenotype analysis, including exploring the potential causal associations between two BD subtypes and lithium responses and comparing the difference in genetic structures among four different psychiatric traits.

**Results:** SNP-level evidence revealed three genomic loci, *SLC25A17, ZNF184*, and *RPL10AP3*, shared by BD I and II, and one locus (*MAD1L1*) and significant gene sets involved in calcium channel activity, neural and synapsed signals that distinguished two subtypes. TWAS data implicated different genes affecting BD I and II through expression in specific brain regions (nucleus accumbens for BD I). Cross-phenotype analyses indicated that BD I and II share continuous genetic structures with schizophrenia and major depressive disorder, which help fill the gaps left by the dichotomy of mental disorders.

**Conclusion:** These combined evidences illustrate genetic convergence and divergence between BD I and II and provide an underlying biological and trans-diagnostic insight into major psychiatric disorders.

## Introduction

Bipolar disorder (BD) is one of the most severe psychiatric disorders, characterized by mood state fluctuation. As one of the top causes of disability worldwide, BD affects more than 40 million people worldwide with a lifespan prevalence of 1%∼4% (Merikangas et al., 2011; Huang et al., 2019), early-onset in adolescents, and elevated risk of suicide (GBD 2016 Disease and Injury Incidence and Prevalence Collaborators, 2018).

Population and molecular studies have proved evidence of the complex etiology of BD. Twin and family studies have estimated that the heritability of BD is over 70% (Edvardsen et al., 2008; Bienvenu et al., 2011). GWAS have brought a deeper insight into BD (Stahl et al., 2019; Li et al., 2021) compared with previous population genetics studies (Bertelsen et al., 1977; Kieseppa et al., 2004). The largest-scale GWAS of BD was recently processed by the Psychiatric Genomic Consortium Bipolar Disorder Working Group (PGC BD) (Mullins et al., 2021), and 64 genome-wide significant loci were identified. However, it failed to display increasing single nucleotide polymorphism (SNP)–level heritability (h^2^_SNP_) of BD (Psychiatric 2011; Mullins et al., 2021).

BD can be categorized into several major subtypes: BD type I (BD I) and type II (BD II), cyclothymia, and other specified bipolar and related disorders, according to the Diagnostic and Statistical Manual Disorders, Fifth Edition (DSM-5). BD I requires manic episodes at least once despite depression states, and BD II is defined as more than one depressive and hypomanic state. The worldwide lifetime prevalence of BD I (0.4%–1.2%) (Bebbington and Ramana 1995; Merikangas et al., 2011; Huang et al., 2019) differs from BD II (0.1%–2.5%) (Bebbington and Ramana 1995; Merikangas et al., 2011; Huang et al., 2019). In addition, clinical presentations and severity vary in the two subtypes (Judd et al., 2008; Merikangas et al., 2011); however, their genetic differences remained unclear due to insufficient sample size or substandard clinical classification (Charney et al., 2017; Bipolar Disorder and Schizophrenia Working Group of the Psychiatric Genomics Consortium, 2018).

Under the dichotomic diagnostic system, BD is hard to be distinguished from schizophrenia (SCZ) and major depressive disorder (MDD). BD with psychotic symptoms or manic BD performs and behaves similarly to SCZ; and oppositely, depressive BD is always misdiagnosed as MDD, resulting in limited therapeutic effects. Additionally, second-generation antipsychotics play an important role in the treatment of BD, SCZ, and MDD, indicating potential shared biological mechanisms among these phenotypes. Linkage disequilibrium (LD) score analysis indicated that BD I is much more genetically correlated with SCZ, whereas the genetic correlation of BD II with MDD is higher (Mullins et al., 2021). These provide a new perspective on the genetic correlation between BD I, BD II, SCZ, and MDD. Moreover, molecular genetic studies have uncovered overlapped risk factors between the genomic architecture of psychiatric disorders (Psychiatric 2011; Charney et al., 2017; Stahl et al., 2019; Mullins et al., 2021); however, current diagnostic systems failed to elucidate it clearly.

To better understand BD etiology and taxonomy, our study aimed to provide more evidence through post-GWAS analyses. Integrative Omics approaches were adopted to navigate functional genes expressed in the influenced brain regions. Additionally, we explored cross-phenotype genetic structure in adult psychiatric disorders. We are trying to enrich the Research Domain Criteria (RDoC) and re-evaluate and provide new evidence for the cross-disease diagnosis of mental disorders.

## Methods and Materials

### Study Design and GWAS Data Resources

**FIGURE 1:**
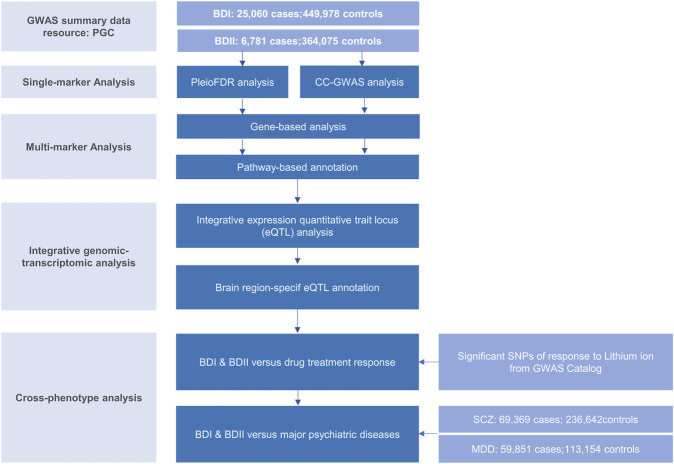
Workflow of key methodological steps in this study. PGC: psychiatric genomics consortium, BD I: bipolar disorder type I, BD II: bipolar disorder type II, SCZ: schizophrenia, MDD: major depressive disorder.

The genome-wide association (GWA) meta-analysis summary data of BD I and II were from the PGC BD, containing BD I (25,060 cases and 449,978 controls) and BD II (6,781 cases and 364,075 controls), respectively. All participants were of European descent, and the diagnosis was explicitly based on international consensus criteria (DSM-IV, ICD-9, or ICD-10). Details on participant and cohort information and quality control can be accessed at Consortium (2020). Nominal significant instrumental variants (IVs) of response to lithium salt in bipolar disorder (Hou et al., 2016b; Song et al., 2016; International Consortium on Lithium et al., 2018) were downloaded from National Human Genome Research Institute–European Bioinformatics Institute (NHGRI-EBI) GWAS Catalog (Buniello et al., 2019) (https://www.ebi.ac.uk/). The latest and biggest GWA meta-analysis summary statistics for SCZ (53,386 cases and 77,258 controls) (Trubetskoy et al., 2022) and MDD except samples in the 23andMe dataset (59,851 cases and 113,154 controls) (Wray et al., 2018) were from PGC website. Non-rsID SNPs were converted using ANNOVA (Wang et al., 2010), and those without rsID were removed. Beta was computed by log (OR). The overall post-GWAS analysis pipeline is shown in Figure 1.

### Single-Marker Analysis

#### PleioFDR Analysis

For genetic overlap, we used pleiotropy-informed conditional false discovery rate (pleioFDR) methods (Andreassen et al., 2013), including conditional FDR (condFDR), an extension of the standard FDR, and conjunctional FDR (conjFDR) analysis, defined in turn as the maximum of the two condFDR values. The pleioFDR provided a conservative estimate of the FDR associated with both phenotypes and was applied to identify specific shared loci. Based on an empirical Bayesian statistical framework, this statistical framework increased statistical power in detecting SNPs that did not reach genome-wide significance. Independent significant SNPs were defined with condFDR <0.01.

#### CC-GWAS Analysis

For genetic uniqueness, we applied the CC-GWAS method (Peyrot and Price, 2021). CC-GWAS perceives differences in minor allele frequencies (MAF) across two traits by analyzing case-control GWAS summary statistics for each other. It weighted the effect size using two methods, ordinary least squares (OLS) weights and exact weights. To avoid suspect null–null SNPs, SNPs with p_OLS_ <5 × 10^−8^ were excluded. Then, those that failed to pass the required level of significance of CC-GWAS (p_EXACT_ >1 × 10^−4^) were excluded to effectively control the type I error rate caused by suspect stress test SNPs.

### Multi-Marker Analysis

#### Gene-Based Analysis

Independent genomic loci were mapped by shared and trait-specific SNPs from GWAS summary data of BD I (PGC), BD II (PGC), and CC-GWAS (this study) using ANNOVAR employed in functional mapping and annotation of GWAS [FUMA (Watanabe et al., 2017)] online platform (https://fuma.ctglab.nl/). Significant SNPs were first selected by LD *r* ^2^ > 0.6 within a 10 kb window. Second, we narrowed lead SNPs with LD *r* ^2^ > 0.1 with the same window. Genomic risk loci were identified by merging lead SNPs if they were closer than 250 kb, thus, containing multiple lead SNPs. The European samples retrieved from the 1,000 Genomes Project phase 3 (1000G EUR) (Genomes Project et al., 2015) were used to calculate LD. To further define independent genomic loci diverged in BD I and II, we utilized MAGMA v1.6 implemented in FUMA (Watanabe et al., 2017). Gene locations and boundaries were from the NCBI Build GRCh37 assembly.

#### Pathway-Based Annotation

Functional annotation was performed to uncover the likely biological mechanisms linking and distinguishing BD I and II. Enrichment for the genes mapped to all (candidate, genes nearest to lead and lead) SNPs in the identified shared loci was evaluated by the Molecular Signatures Database (MsigdB) *via* a hypergeometric test implemented in FUMA (Watanabe et al., 2017). Genes without unique Entrez ID or pathways containing less than two genes were removed. The results were adjusted by Benjamini–Hochberg false discovery rate (BH FDR) of 0.05.

### Integrative Genomic-Transcriptomic Analysis

#### Integrative Expression Quantitative Trait Locus Analysis

To detect important but non-genome-wide significant sites, we first used summary-data–based Mendelian randomization (SMR) (Zhu et al., 2016) to estimate loci with strong evidence of causal effects of blood [eQTLGen Consortium (Vosa et al., 2021), 31,684 whole blood samples] and a large-scaled meta-data for brain resources [GTEx Consortium et al., 2017; Qi et al., 2018), CMC(Fromer et al., 2016) and ROSMAP (Ng et al., 2017), 1194 estimated effective sample] *via* gene expression in BD I and BD II risk. SMR analysis was limited to significant cis eQTL (p_eQTL_ < 5 × 10^−8^), with MAF >0.20, and passing heterogeneity in dependent instruments outlier (HEIDI-outlier) test (*p* ≥ 0.01) due to its conservativeness (Zhu et al., 2016; Zhu et al., 2018). Significant loci were filtered after multiple testing and within 1 MB distance from each probe.

#### Brain Region-specific eQTL Annotation

Likewise, we conducted brain-specific analyses using e-MAGMA (Gerring et al., 2021) and FUSION(Gusev et al., 2016), transcriptome-wide association studies (TWAS) to map genes based on precomputed tissue-specific eQTL statistics leveraging 13 brain tissues of GTEx v8 (Consortium 2020) and test whether SNPs influencing gene expression are associated with BD I and II. The 1000G EUR were used as a reference dataset to account for LD between SNPs. FDR correction was also applied to control the multiple tests performed on the numbers of genes in each process.

### Cross-Phenotype Analysis

#### BD I and II Versus Lithium Treatment Responses

We then investigate bi-directional causal relationships between BD I and II and lithium salt response using the “two-sample MR” (Version 0.5.3) and “Mendelian randomization” (Version 0.5.1) R packages. We selected independent SNPs with *p*-value < 5 × 10^−6^ and harmonized exposure and outcome data by removing SNPs with large MAF differences or different reference alleles. For two-sample MR analysis, we used inverse variance weighted (IVW), weighted median, and MR-Egger as primary methods. MR-Egger intercept test and MR pleiotropy residual sum and outlier (MR-PRESSO) test (Verbanck et al., 2018) were used to evaluate potential horizontal pleiotropy.

#### BD I and II Versus Major Psychiatric Diseases

To explore genetic causal associations between BD I, BD II, SCZ, and MDD, we conducted a bi-directional MR analysis between each pair of the heritable variables using generalized summary-data–based Mendelian randomization (GSMR) (Yang et al., 2011). The estimated effect size and its standard error from multiple instrumental variants were associated with the exposure trait at a genome-wide significant level (*p* < 5 × 10^−8^). Attribute to insufficient instruments included in analyses, a *p*-value threshold of 5 × 10^−5^ was used. In GSMR, genetic instruments with pleiotropic effects are detected and eliminated by the HEIDI-outlier procedure, the same with SMR. We used default options in GSMR with HEIDI testing for the detection of instrumental outliers (LD *r* ^2^ < 0.05, and at least 10 SNPs were required).

Finally, MiXeR (Frei et al., 2019; Holland et al., 2020) was applied as a polygenic overlap analysis. Univariate models estimated polygenicity (estimated number of variants) and discoverability (the average magnitude of additive genetic associations across variants) of each phenotype. Bivariate Gaussian mixture models were also applied to estimate the number of variants influencing each trait that explained 90% of h^2^_snp_ and their overlap with each other. MiXeR calculated a Dice coefficient, a ratio of shared variants to the total number of variants, to evaluate the polygenic overlap. In line with the Akaike information criterion (AIC), MiXeR evaluated model fitting based on the current power of input summary statistics.

## Results

### Genetic Overlaps Between Bipolar Type I and II

**FIGURE 2:**
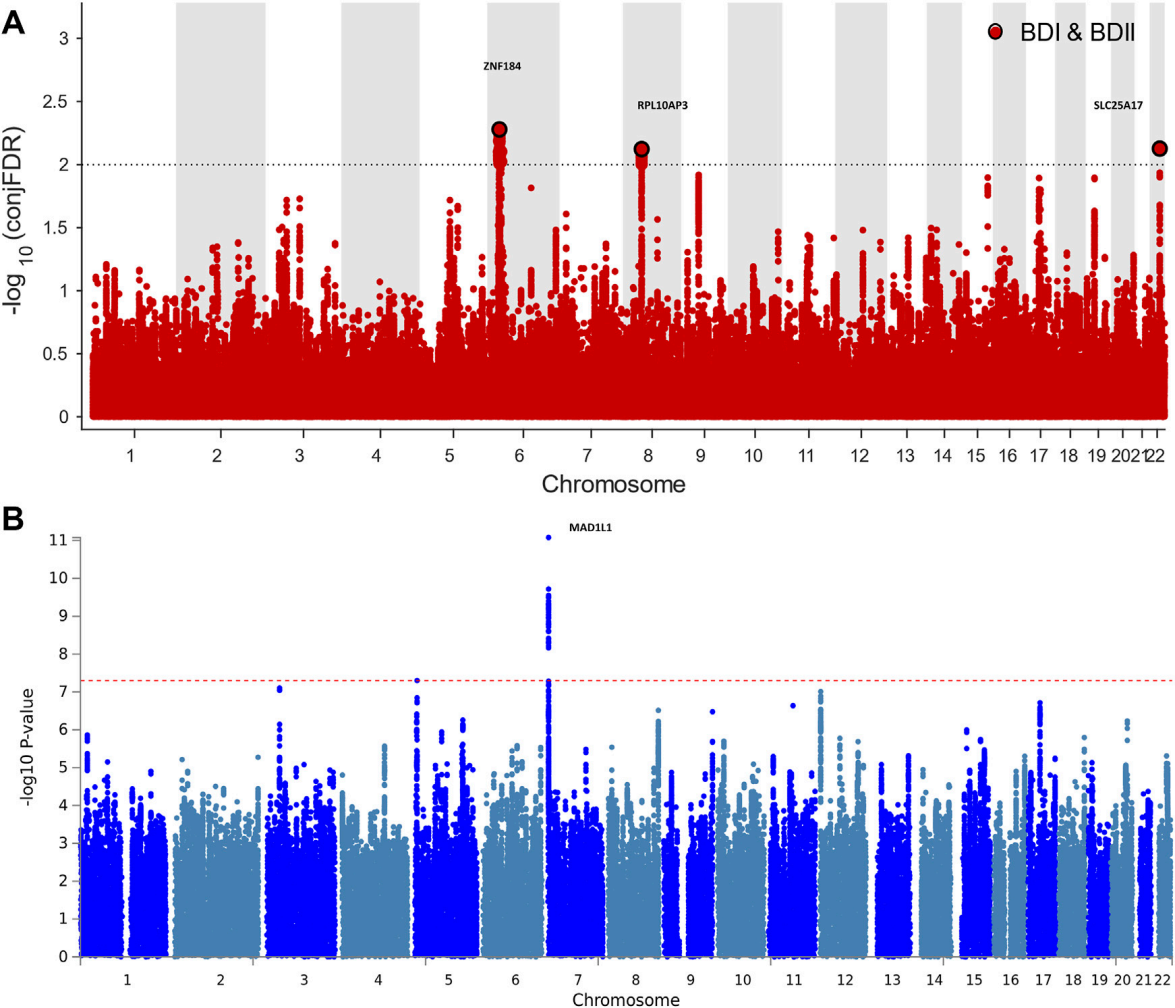
Manhattan plots showing the association statistics for single marker analysis of BD I and II genetic overlap (Figure 2A) and distinctness (Figure 2B). The *y*-axis shows the GWAS −log10 *p*-values per SNP across chromosomes 1-22. **(A)** SNPs with conditional *p*-value < 1 × 10^−2^ are shown with large black points. **(B)** SNPs identified by case–case genome-wide association analysis (CC-GWAS) with *p* < 5 × 10^−8^. The figures show the localization of significant loci. Details about the loci are provided in Tables 1 and 2.

For signals shared by BD I and BD II, the conjFDR analysis identified 74 significant SNPs (*p* < 0.01) that are mapped to three genomic loci (Table 1; Supplementary Table S1; Figure 2A): *ZNF184* (zinc finger protein 184), mapped by *rs67240003* (ALT:T, REF:G, MAF:0.044, p_FDR_ = 5.26 × 10^−3^) and *RPL10AP3* (ribosomal protein L10a pseudogene 3)*,* mapped by rs6990255 (ALT:T,REF:C, MAF:0.042, p_FDR_ = 7.54 × 10^−3^). The third one is consistent with the newest BD GWAS: *SLC25A17* (solute carrier family 25 member 17), mapped by rs5758064 (ALT:C, REF:T, MAF:0.49, p_FDR_ = 7.47 × 10^−3^). The shared genomic loci, candidate independent SNPs, allelic association, and novelty for BD are summarized in Supplementary Table S1. The stratified conditional quantile–quantile (Q–Q) plots showed SNP enrichment for BD I condition on association with BD II and vice versa (Supplementary Figure S1), suggesting the existence of polygenic overlap.

**TABLE 1:** Conjunction FDR; pleiotropic loci in BD type I (BD I) and BD type II (BD II) (BD I & BD II) at conjFDR <0.01.

Locus	CHR	SNP	Position	Neighbor gene	A1	A2	ConjFDR BD I&BD II	Zscore_BD I	Zscore_BD II
1	6p22.1	rs67240003	27443202	ZNF184	T	G	0.00526327	−4.74249	−4.3186
2	8p12	rs6990255	34126948	RPL10AP3	T	C	0.0075384	4.350196	4.21441
^a^3	22q13.2	rs5758064	41153879	SLC25A17	C	T	0.00747462	−4.74249	−4.21714

a Same locus identified in previous BD genome-wide association studies.

Independent complex or single gene loci (*r* ^2^ < 0.2) with SNP(s) with a conjunctional FDR (conjFDR) < 0.05 in BD I and BD II. All SNPs with a conjFDR value of 0.05 (bidirectional association) and association with BD I & BD II are listed and sorted in each LD block. We defined the most significant SNP in each LD block based on the minimum conjFDR. Chromosome (CHR), minor allele (A1), and major allele (A2), Z-scores for each pleiotropic locus are provided. All data were first corrected for genomic inflation. Locus name is based on exonic lead SNPs. Remaining locus name is based on the nearest gene and does not refer to any inferred biological function. Details are in Supplementary Table S1.

### Genetic Distinction of Bipolar Type I and II

CC-GWAS analysis was applied to the publicly available summary statistics for BD I and BD II. The only one CC-GWAS BD I versus BD II SNP was *rs12154473* (ALT:G, REF:A, MAF: 0.56), mapping *MAD1L1* (mitotic arrest deficient 1 like 1*,* p_OLS_ = 2.83 × 10^−8^; p_EXACT_ = 6.07 × 10^−5^; Table 2; Supplementary Tables S2 and S3; Supplementary Figure S2). The Manhattan plot of CC-GWAS results is shown in Figure 2B.

**TABLE 2:** Distinguished loci between BD I and BD II by CC-GWAS results at OLS <5 × 10^−8^ and EXACT <1 × 10^−4^.

Disorders	SNP	CHR	Position	Locus	A1B1(OLS)			A1B1 (Exact)		
					Beta	Se	*p*	Beta	Se	*p*
BD I & BD II	rs12154473	7	1982181	MAD1L1	0.0117	0.00211	2.83E-08	0.0435	0.0108	6.07E-05

For the CC-GWAS–specific locus, the lead CC-GWAS SNP and its chromosome, physical position, locus name, respective case–control effect sizes and *p*-values and the CC-GWAS OLS and exact case–case effect size, standard error(se), and p‐value. Details are in Supplementary Table S3.

In the gene-based analysis, CC-GWAS displayed nine significant genes between BD I and II after multiple testing (*p* < 0.05/18,626 = 2.68 × 10^−6^). A total of 129 genes were significant for BD I (*p* < 0.05/18,847 = 2.65 × 10^−6^). *CACNA1C* (gene calcium voltage-gated channel subunit alpha1 C*, p* = 2.80 × 10^−11^), *MAD1L1* (*p* = 7.56 × 10^−11^), and *TMEM258* (transmembrane protein 258*, p* = 9.48 × 10^−11^), were the top three genes of BD I. The only significant gene of BD II (*p* < 0.05/18,830 = 2.66 × 10^−6^) was slit guidance ligand 3 (*SLIT3, p* = 7.92 × 10^−9^) (Supplementary Tables S4 and S12).

In the pathway analysis, 11, 6 and 1 pathways were significantly enriched by the genes through MAGMA analysis for BD I, BD II, and CC-GWAS summary statistics (p_Bonferroni_ < 0.05), respectively. As for BD I, the “neuron part”, “somatodendritic compartment”, “high voltage-gated calcium channel activity”, and “voltage-gated calcium channel activity involved in cardiac muscle cell action potential” were gene ontology pathways verified by CC-GWAS and BD I. As for BD II, the only significant pathway was the “Hirsch cellular transformation signature up” (*p* = 9.84 × 10^−5^) (Supplementary Table S5).

### TWAS Analyses in Blood and Brain Regions

In the SMR process, 11 in brain (p_SMR_ < 6.61 × 10^−6^) and 49 in blood (p_SMR_ < 3.19 × 10^−6^) putative BD I-associated genes were identified after multiple testing corrections and a heterogeneity test. The top loci were *NMB* (neuromedin B) and *FADS1* (fatty acid desaturase 1) for BD I in blood and brain, respectively (Supplementary Figure S3). We did not observe significant results for BD II after multiple testing. The related genomic loci, candidate SNPs, and allelic association for BD I are summarized in Supplementary Tables S6 and S7.

In the brain region-specific TWAS analysis, e-MAGMA identified 148 loci (p_FDR_ <0.05) of BD I. *FADS1* (p_FDR_ = 1.87 × 10^−7^), *PLEC* (plectin*,* p_FDR_ = 3.10 × 10^−7^), and *ITIH4* (inter-alpha-trypsin inhibitor heavy chain 4*,* p_FDR_ = 3.10 × 10^−7^) were the top ones. These genes encompassed three brain regions, including the hypothalamus, amygdala, and cerebellum (Supplementary Table S8). Similarly, FUSION indicated 336 genes (p_FDR_ <0.05, 9 hits of previous GWAS of BD achieved nominal significance) of BD I (Supplementary Table S9) among all the 13 brain regions.

**FIGURE 3:**
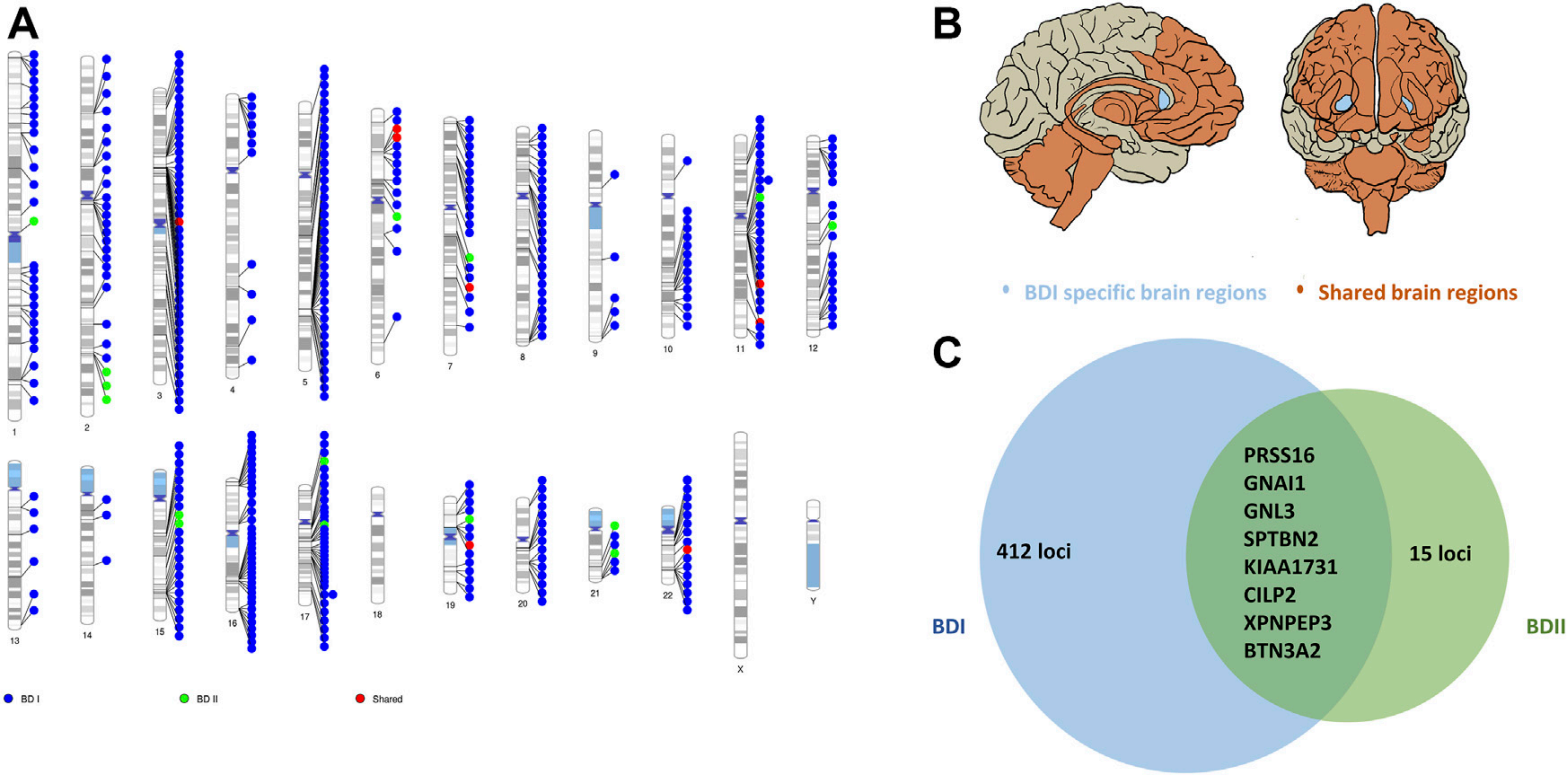
Shared and trait-specific eQTLs of BD I and BD II. **(A)** Genomic regions that are specific to BD I (blue points), specific to BD II (green points), and shared (red points). **(B)** Associated 13 brain regions with BD I and BD II: cortex, frontal cortex, anterior cingulate cortex, caudate, putamen, hypothalamus, amygdala, hippocampus, substantia nigra, cerebellum, cerebellar hemisphere, spinal cord cervical c-1 (orange area), and BD I specific brain region: nucleus accumbens (light blue area). **(C)** Prioritized genes for shared and trait-specific regions. Genes presented reached significance (after FDR correction) in the TWAS test and were identified by two methods (FUSION and e-MAGMA). The numbers represent trait-specific genes identified by at least one method. Details are in Supplementary Tables S8–S12.

As for BD II, two genes showed significant association with BD II by e-MAGMA after correction for multiple testing: *GNAI1* (G protein subunit alpha I 1 on chromosome 7, p_FDR_ = 2.64 × 10^−7^) and *PRSS16* (serine protease 16 on chromosome 6, p_FDR_ = 1.40 × 10^−5^). Both were over-expressed in the cerebellum (Supplementary Table S10). A total of 21 genes were significant by FUSION after multiple tests among brain regions without nucleus accumbens (NAc) (Supplementary Table S11). The top hit was AC005932.1 (p_FDR_ = 2.07 × 10^−4^), a novel nominal significant gene for BD II (Figure 3; Supplementary Figures S4 and S5; Supplementary Table S12).

### Correlations Between BD I and II and Response to Lithium

We selected 16 SNPs from the GWAS catalog mapped to “response to lithium ion”. After selection and harmonization, ten SNPs were included in the final bi-directional Mendelian randomization between BD I and response to lithium ion. The estimate effect was positive and statistically significant in weighted median [beta = 1.89; standard error (SE) = 0.426; *p* = 9.57 × 10^−6^] but not significant in IVW (beta = 2.22; SE = 1.66; *p* = 0.180). Nine SNPs were included in the analysis between BD II and response to lithium ion, showing negative results in IVW (beta = 3.27; SE = 2.28; *p* = 0.153) or weighted median (beta = 3.29; SE = 2.21; *p* = 0.136), even estimates were higher compared to BD I. Based on different hypotheses of whether there is a dose-response relationship between the shared genetic instruments of exposure and outcome with an intercept versus whether omitting each genetic variant from analysis differs from the original model, MR-Egger and MRR-PRESSO displayed similar results for the heterogeneity test. The relationship between BD I and response to lithium failed to pass MR-Egger intercept analysis (MR-Egger intercept = 0.39; *p* = 0.01) or MR-PRESSO (p_Global_ test <0.001), indicating the possible existence of horizontal pleiotropy (Supplementary Table S13; Supplementary Figure S6).

### Genetic Overlaps Between BD I and II and Other Traits

GSMR analyses provided evidence that genetically SCZ provided a 0.50-fold and 0.32-fold causality increase in BD I and BD II, respectively. Inversely, MDD increased causality with BD I and BD II by 0.23-fold and 1.11-fold, respectively. In the other direction, BD I provided 0.32-fold causal effect on SCZ (beta = 0.32, SE = 0.019, *p* = 3.70 × 10^−62^), comparing with less effect on MDD (beta = 0.043, SE = 0.018, *p* = 1.36 × 10^−2^). Since the number of SNPs ought to be over 10, *p*-value threshold was set to be 5 × 10^−5^ when BD II was computed into clumping as exposure trait. The causal relationship between BD II and SCZ (beta = 0.073, SE = 0.008, *p* = 1.12 × 10^−21^) were very close to BD II and MDD (beta = 0.050, SE = 0.008, *p* = 7.71 ×× 10^−11^). BD I (beta = 0.505, SE = 0.044, *p* = 5.18 ×× 10^−31^) and BD II (beta = 0.173, SE = 0.012, *p* = 2.78 ×× 10^−50^) were presented with causality with each other (Supplementary Table S14).

MiXeR estimated that approximately 7.88 k (SE = 0.26 k) variants influence BD I, which was comparable to the case of SCZ (9.82 k; SE = 0.22 k), lower than that for major depression (21.6 k, SE = 2.64 k) and 19.82 k (SE = 21.12 k) variants influenced BD II. The deficiency of sample size may explain the odd statistics in BD II. MiXeR also revealed a higher polygenicity in BD II and MDD than in BD I and SCZ. In BD I and BD II, 7.47 k (SE = 0.29 k) and 7.47 k (SE = 1.73 k) variants were associated with SCZ; 5.44 k (SE = 0.59 k) and 13.23 k (SE = 5.91 k) variants were associated with MDD, respectively. Consistent with LD score regression, MiXeR showed that BD I enjoyed higher genetic overlap with SCZ (rg = 0.70) than with MDD (rg = 0.39), and oppositely, BD II possessed higher genetic overlap with MDD (rg = 0.68) than with SCZ (rg = 0.61) (Supplementary Figure S7; Supplementary Table S15).

## Discussion

The present study is the first comprehensive post-GWAS analysis of BD I and II using the largest BD dataset (Mullins et al., 2021). Different from the original study that aimed to identify novel genes and drug targets using overall BD as the primary phenotype (Mullins et al., 2021), our integrative genomic analyses directly answered the following question: what are the shared and distinct genetic components of BD subtypes? In this study, we corroborated and expanded evidence from previous clinical and genetic studies that there did exist a partially shared genetic basis between BD I and II and provided further insights into their genetic divergence. When compared to other earlier studies (Schizophrenia Working Group of the Psychiatric Genomics Consortium, 2014; Charney et al., 2017; Huckins et al., 2017; Mullins et al., 2021) on the same research question (Supplementary Table S16), our study is innovative from different aspects: 1) largest sample size for BD I and II; 2) more systematic statistical genetics analyses within BD itself and across major psychiatric disorders; and 3) new biological explanation to the distinction of BD subtypes.

For genetic convergence, all of these loci identified shared by BD I and II were previously reported to be associated with bipolar disorder, depression, ADHD, autism spectrum disorder, or schizophrenia (Cross-Disorder Group of the Psychiatric Genomics 2013; Autism Spectrum Disorders Working Group of The Psychiatric Genomics Consortium, 2017; Ferguson et al., 2018; Wu et al., 2020), underpinning their contribution to mental disorder risk. *ZNF184* has been reported to be likely associated with subcortical volume (van der Meer et al., 2020), indicating a potential biological function in BD neurodevelopment (Valli et al., 2019). Research for links between mental and physical disorders is also proposed (Van Veldhoven 2010; Liu et al., 2017; Wain et al., 2017; Ferguson et al., 2018).

For genetic divergence, notably, *MAD1L1*, which was reported to be genome-wide significant in the two previous BD GWAS (Hou et al., 2016a; Ikeda et al., 2018) in Asian samples, nominally distinguished BD I and II in this study. This gene contributes to cell cycle control through the regulation of mitosis and has been shown to have a pleiotropic effect on psychosis and BD (Ruderfer et al., 2014; Schizophrenia Working Group of the Psychiatric Genomics Consortium, 2014; Charney et al., 2017). Moreover, *MRM1* (mitochondrial rRNA methyltransferase 1)*, ZNHIT3* (zinc finger HIT-type containing 3)*, DHRS11* (dehydrogenase/reductase 11)*,* and *GGNBP2* (gametogenetin binding protein 2) were first reported as significant in the gene-based test. *SLIT3* was identified to be BD II-specific by gene-based analysis. *SLIT3* was reported to increase schizophrenia susceptibility (Shi et al., 2004). Population difference (EUR vs. Han) and phenotype heterogeneity across countries may explain this interesting observation. *SLIT3* has also been shown to play a critical role in the formation and maintenance of the nervous system (Vargesson et al., 2001), indicating a generally shared genetic association among psychiatric disorders. However, it was not discovered to be associated with BD I, which indicated *SLIT3* could be a marker to distinguish BD II.

Enriched gene-sets of BD I were involved in neuronal and postsynaptic compartments as well as calcium channel activity, triggering presynaptic signaling, which reconfirmed cross-phenotype correlation across BD, SCZ, ASD, and cognitive deficiency (Pescosolido et al., 2013; Cupertino et al., 2016; Forstner et al., 2017; Bipolar, Schizophrenia Working Group of the Psychiatric Genomics Consortium. Electronic address et al., 2018). These pathway processes indicate that BD I primarily represents BD biological features and point to deeper research into common biological pathogenesis among mental disorders. In comparison, the BD II-specific pathway effects are generally linked with cancer and inflammatory and metabolic diseases (Hirsch et al., 2010), suggesting that larger cohorts are required to provide a mechanic prompt for further research on BD II.

Interestingly, from the integrative Omics analysis, we found that *FADS1*, one of the three top eQTL-associated loci shared by both brain and blood, is presented with opposite directions of effect on gene expression in the two different tissues. This observation also suggests that *FADS1* possibly plays a role in the tissue-specific gene regulation of BD I. The possible reason was that *FADS1* is strongly associated with blood cell and lipid and glucose metabolite (Sabatti et al., 2009; Tintle et al., 2015; Tabassum et al., 2019; Chen et al., 2020),and is, thus, highly expressed in blood. Brain region-specific eQTL analysis yielded 15 genes specific for BD II. These eQTLs provide promising candidate genes for subsequent functional experiments, especially *NOS2* (Nitric oxide synthase 2) and *CASP8* (Caspase-8), participate in drug metabolism (Whirl-Carrillo et al., 2012), despite no correlation to psychosis was yet found. While several of these genes are implicated by genome-wide significant loci, many of them are not the closest gene to the index SNP, highlighting the value of probing underlying molecular mechanisms to prioritize the most likely causal genes in each corresponding locus and moving from genes to functional mechanism. However, most genes are not overlapped among gene-based and TWAS analyses due to different hypotheses on how SNPs affect gene expression (Supplementary Table S12).

In addition, BD I and BD II significantly differ in biosignatures as revealed by gene expression differentiation in functional brain regions and drug response in this study. Gene expression differentiation in NAc might represent an endophenotype of BD I addressing dysfunction of brain circuits. By regulating dopamine release and the midbrain dopamine system, NAc contributes to the onset of SCZ (Eastwood et al., 2005; Kozlovsky et al., 2006), especially for delusion and hallucination. It is also a contributor to the pathophysiology of BD, as shown in a postmortem brain analysis (Kunii et al., 2019). Even though we did not find direct causal relationships between BD I, BD II, and lithium response, there indeed exists a linkage with lithium response following the guidelines (Yatham et al., 2018): lithium was first-line to BD I, but not to BD II. Lithium is more effective for patients sharing etiological homogeneity; based on longitudinal stability and familial clustering, lithium response has been suggested to define a distinct genetically based BD nosology (International Consortium on Lithium et al., 2018). Therefore, biological indicators such as treatment response, clinical prognosis, and progression of BD I and BD II should be included in genetic analysis to enable improved precise clinical decision-making. It is also the RDoC standard that a combination of neuroscience research will be helpful for future genetic research, even altering clinical management (Insel et al., 2010).

Another interesting finding of this study is that, despite bidirectional causal associations from GSMR and mixed directional overlap from MiXeR, whether the causal relationships driven by other covariates (Yang et al., 2011) is unclear, and meanwhile, MiXeR analysis prompted a high clinical heterogeneity for BD I-BD II pair, when compared with BD I-SCZ or BD II-MDD pair. One explanation could be that BD I and II may help fill the gap across mental disorders by revealing transdiagnostic biotypes (Cross-Disorder Group of the Psychiatric Genomics Consortium. Electronic address and Cross-Disorder Group of the Psychiatric Genomics 2019). Insights into such continuous genetic structure, rather than completely independent disease entities, may greatly contribute to clinical decision-making in prophylaxis or management of the disorder. The findings of this study will also trigger larger studies on BD II and other biotypes, such as psychosis bipolar disorder and cyclothymia, because current BD GWAS mainly reflected the genetic characteristics of BD I, the majority of overall BD cases. Although a large sample size of GWAS is always important in nowadays genomic studies, the statistical power will be greatly reduced when there is nonnegligible clinical heterogeneity caused by the classification system within disease phenotype (Mitchell et al., 2021). Therefore, as RDoC emphasized, large-scale transdiagnostic investigations are urgently needed to untangle whether impairment or symptoms can be regarded as subtype-specific, and so do multi-omics analysis (Cuthbert 2020).

One of the potential limitations of our study is that the population imbalance of BD I and BD II GWAS may be susceptible to reduced power; however, it did not lead to inflated type I error in our post-GWAS analysis. Another limitation is that we failed to achieve individual genotypes, leading to the incompleteness of some important analyses, such as polygenic risk score (PRS) calculation. Finally, our study only obtained GWAS summary statistics of BD I and BD II, lacking data from other BD subtypes. Once other characteristics of clinical subtypes (psychotic symptoms) are available in PGC or other groups, further refined genetic architecture for BD should be explored by a more systematic comparison in future.

In summary, genetic evidence deepens our understanding of the biological etiology of BD and prioritizes a set of candidate genes distinguishing BD I and II for functional follow-up experiments and indicates a spectrum connecting psychiatric disorders, which enable better ways to optimize nosology and precise treatments in psychiatry.

## Data Availability

The data that support the findings of this study were derived from the following resources available in the public domain: https://www.med.unc.edu/pgc/download-results/.

## Abbreviations

BD, Bipolar disorder; BDI, Bipolar disorder type I; BDII, Bipolar disorder type II; condFDR, Conditional false discovery rate; conjFDR, Conjunctional false discovery rate; eQTL, Expression quantitative trait locus; FUMA, Functional mapping and annotation of GWAS; GSMR, Generalized summary-data–based Mendelian randomization; GWAS, Genome-wide association study; HEIDI, Heterogeneity in dependent instruments; IVs, Instrumental variants; IVW, Inverse variance weighted; LD, Linkage disequilibrium; MAF, Minor allele frequency; MAGMA, Multi-marker analysis of genomic annotation; MDD, Major depressive disorder; MR-PRESSO, MR pleiotropy residual sum and outlier; NHGRI-EBI, National Human Genome Research Institute–European Bioinformatics Institute; OLS, Ordinary least squares; PGC, Psychiatric Genomics Consortium; pleioFDR, pleiotropy-informed conditional false discovery rate; RDoC, Research Domain Criteria; SCZ, Schizophrenia; SMR, Summary-data–based Mendelian randomization; SNP, Single nucleotide polymorphism; TWAS, Transcriptome-wide association analyses.
